# On Leveraging Machine Learning in Sport Science in the Hypothetico-deductive Framework

**DOI:** 10.1186/s40798-024-00788-4

**Published:** 2024-11-14

**Authors:** Jordan Rodu, Alexandra F. DeJong Lempke, Natalie Kupperman, Jay Hertel

**Affiliations:** 1 Department of Statistics, University of Virginia, Charlottesville, VA USA; 2 Department of Physical Medicine and Rehabilitation, School of Medicine, Virginia Commonwealth University, Richmond, VA USA; 3 School of Data Science, University of Virginia, Charlottesville, VA USA; 4 Department of Kinesiology, University of Virginia, Charlottesville, VA USA

## Abstract

**Abstract:**

Supervised machine learning (ML) offers an exciting suite of algorithms that could benefit research in sport science. In principle, supervised ML approaches were designed for pure prediction, as opposed to explanation, leading to a rise in powerful, but opaque, algorithms. Recently, two subdomains of ML–explainable ML, which allows us to “peek into the black box,” and interpretable ML, which encourages using algorithms that are inherently interpretable–have grown in popularity. The increased transparency of these powerful ML algorithms may provide considerable support for the hypothetico-deductive framework, in which hypotheses are generated from prior beliefs and theory, and are assessed against data collected specifically to test that hypothesis. However, this paper shows why ML algorithms are fundamentally different from statistical methods, even when using explainable or interpretable approaches. Translating potential insights from supervised ML algorithms, while in many cases seemingly straightforward, can have unanticipated challenges. While supervised ML cannot be used to replace statistical methods, we propose ways in which the sport sciences community can take advantage of supervised ML in the hypothetico-deductive framework. In this manuscript we argue that supervised machine learning can and should augment our exploratory investigations in sport science, but that leveraging potential insights from supervised ML algorithms should be undertaken with caution. We justify our position through a careful examination of supervised machine learning, and provide a useful analogy to help elucidate our findings. Three case studies are provided to demonstrate how supervised machine learning can be integrated into exploratory analysis. Supervised machine learning should be integrated into the scientific workflow with requisite caution. The approaches described in this paper provide ways to safely leverage the strengths of machine learning—like the flexibility ML algorithms can provide for fitting complex patterns—while avoiding potential pitfalls—at best, like wasted effort and money, and at worst, like misguided clinical recommendations—that may arise when trying to integrate findings from ML algorithms into domain knowledge.

**Key Points:**

- Some supervised machine learning algorithms and statistical models are used to solve the same problem, *y = f(x) + ε*, but differ fundamentally in motivation and approach.
- The hypothetico-deductive framework—in which hypotheses are generated from prior beliefs and theory, and are assessed against data collected specifically to test that hypothesis—is one of the core frameworks comprising the scientific method. In the hypothetico-deductive framework, supervised machine learning can be used in an exploratory capacity. However, it cannot replace the use of statistical methods, even as explainable and interpretable machine learning methods become increasingly popular.
- Improper use of supervised machine learning in the hypothetico-deductive framework is tantamount to p-value hacking in statistical methods.

## Introduction

### The Hypothetico-Deductive Framework

Consider the following scenario: a researcher seeks to identify aspects of movement that are associated with a greater or lesser extent of post-exercise pain. A common strategy to uncover such associations is to model the data as $y=f\left(x\right)+\epsilon$ (think: regression) or some variant depending on the task (think: logistic regression, where $y$ takes on a value of $0$ or $1$, and the regression is specified with log-odds as the outcome, instead of $y$ directly). The function $f\left(x\right)$ can be linear or nonlinear, depending on the need of the investigator and the maturity of domain knowledge. Variables included in a model may be of inherent interest or are included to control for known variation in post-exercise pain research. In a classical statistics approach, the functional form of $f\left(x\right)$ is explicitly described by the scientist in terms of linearity, interactions between variables, and variable inclusion. Coefficients for variables, interactions, and nonlinearities can be examined for their “significance” in the model.

In this manuscript, we consider the scenario above in the context of the hypothetico-deductive reasoning [1, 2] in which hypotheses are generated from prior beliefs and theory, and are assessed against data collected specifically to test that hypothesis. (Other productive methods of reasoning coexist in science (see, e.g., [3] and [4]) but comparing and contrasting methods of reasoning is outside the scope of this manuscript.) In the above scenario, a hypothesis (e.g. a certain aspect of movement is associated with greater post-exercise pain) can be encoded by constructing a model that relates variability in the independent variable under study (aspect of movement) with variation in the dependent outcome (post-exercise pain), while controlling for known factors. After fitting the model, the coefficient (or coefficients depending on the complexity of the model or phenomenon under study) for the variable of interest can be examined to determine if that variable could account for a significant amount of variation in the outcome, and evidence for or against the hypothesis can be added to the literature.

Importantly, this piece of evidence represents one view of the hypothesis under one set of data. Using the same data set, the myriad choices made by the scientist (from cleaning the data, to choice of transformations of the data, to what covariates are included in a model and how they are included) can affect the direction, magnitude, and significance of an effect [5]. In addition, the data set is but a sample from a particular subpopulation. The same effect may not hold in a different subpopulation and must necessarily be subject to a different study [2]. The apparent brittleness of a single study underscores the fact that science is a communal enterprise [6]. Conclusions from a single study are part of a broader effort that examines the hypothesis in different subpopulations and under different modeling choices. Assessing the plausibility of evidence from a particular study, and understanding why two studies might yield different conclusions, requires a high degree of transparency [5, 6], including but not limited to access to the nature of the subpopulation under study, how the data were collected and cleaned, and what modeling choices were made.

### Supervised Machine Learning

Recently, a new set of approaches has gained increasing interest in the scientific community, which are grouped under the umbrella term “machine learning” (ML) [and sometimes, interchangeably, artificial intelligence (AI)]. Perhaps confusingly, a suite of ML algorithms categorized as “supervised learning” [7] can express the prediction problem in a way that is strikingly similar to the one described above: $y=f\left(x\right)+\epsilon$. In principle, ML algorithms solve a “pure prediction” problem [8] and were not explicitly designed for *explanation*. The goal in both the ML and statistical approaches is to find a suitable function $f\left(x\right)$. But they differ in how they do so, the purpose of the function $f\left(x\right)$, and what is considered a suitable function (see Sect. “Explainable and Interpretable Machine Learning Provide (Some) Access to Understanding how an Algorithm Works, but Still Pose Issues in the Hypothetico-Deductive Framework” for an in-depth discussion of these differences).

Machine learning provides a useful and powerful suite of tools for scientific research. For instance, when pure prediction is needed, under suitable circumstances [3, 8–10], ML can provide an ideal solution, though effective implementation is not without its challenges (see, e.g., [11]). We leave debate as to when ML is suitable for prediction in sport science to other papers. This article is forward-facing, and focuses on the use of ML specifically in the hypothetico-deductive framework as described above. While supervised ML is frequently thought of as “black box” [12] due to a typical lack of transparency, the recent rise in interest of interpretable and explainable ML has brought the possibility of gleaning clinical guidelines from supervised ML algorithms into view (see, e.g., [13] for a study that attempts to do this). However, we argue that the ability to interpret or explain a supervised ML algorithm does not summarily merit use for confirming association between variables and an outcome. This work attempts to stave off any misconception that supervised ML algorithms can be a direct alternative to statistical models in the hypothetico-deductive framework, and to propose practical ways in which supervised ML can be useful.

We want to be precise about what this paper is and isn’t addressing. As mentioned, supervised ML was designed for pure prediction problems (which emphasize getting accurate predictions—understanding how algorithms make predictions is a secondary concern that may or may not be of interest), including scenarios involving both regression and classification. Pure prediction plays an important role in sport science, and supervised ML (and indeed all of ML) provides a powerful tool in the toolbox. This paper does not address pure prediction problems. Questions addressing under what circumstances ML should be used for pure prediction are both interesting and important, however we relegate those to another paper (to see why supervised ML may not be appropriate for some pure prediction problems, see for instance [9]).

This paper asks if and how supervised ML can be used to *drive explanation in the hypothetico-deductive framework*. Importantly, prediction plays a role in explanation [3]. For instance, a pure prediction algorithm can help us assess how much room there is under certain data scenarios for theoretical development. If the current state-of-the-art explanatory model offers nearly the same predictive performance as a pure prediction algorithm, this suggests that novel measurements may need to be collected to further understanding in a domain. On the other hand, if the gap between the explanatory model and pure prediction algorithm is wide, this suggests that features (e.g. functions) of the independent variables we are currently collecting *may* (though this is not guaranteed) offer additional explanatory power. This use of prediction is outlined in point 6 of Sect. 1.4 of of [3]. Further, when a prediction gap exists, the pure prediction algorithm may provide insights into what features (e.g. interactions or other nonlinearities, additional variables, etc.) may be useful to consider (see points 1 and 3 in the same section).

In this paper, we argue that translating between insights from pure prediction algorithms and scientifically consistent explanatory models in the hypothetico-deductive framework is not always easy, and that an insight that seems promising in the context of a pure prediction problem may be difficult to study under the hypothetico-deductive framework where consistency with prior studies and transparency are required. In fields where collecting data may be expensive, or where studies can have clinical implications, it is critical to add protections against “leaking” information gleaned from a pure prediction algorithm into the hypothetico-deductive framework without proper contextualization. Adding such protections becomes even more important as explainable and interpretable ML (see Sect. “The Field of Machine Learning can be Divided into Several Overlapping Subdomains. We Briefly Describe Six Subdomains that are Most Relevant to this Paper”) gain popularity. Importantly, where the ability to glean information from an ML algorithm used to rely extensively on the opacity of the algorithm (the LASSO [14] is inherently much more interpretable than a neural network), the rapidly developing field of explainable ML makes it (at least) feel like the gap between the accessibility of interpretable ML algorithms and opaque ML algorithms is narrowing.

In this paper, we provide two such protections that can be implemented which we feel balance the promise of leveraging supervised ML while guarding against potentially wasteful or even harmful efforts. We also describe why, even with inherently transparent algorithms like the LASSO, translating insights to scientifically consistent explanatory models may be challenging. But we caution that this paper should not be construed more broadly. We believe there are many promising ways to take advantage of ML (more broadly) for many scientific pursuits (more broadly) but that each ML subdomain and scientific pursuit pairing should be considered separately. In fact, we do not even intend to close the door on the ways that supervised ML can be used in the hypothetico-deductive framework. However, we hope that this paper provides a blueprint for the types of issues that should be considered when contemplating how to leverage ML in sport science.

The time for addressing these issues is now. A search of Nature and Nature research journals (www.nature.com) returns over 8000 articles mentioning machine learning, deep learning (a specific type of machine learning algorithm), or both, from the previous 12 months alone (search date 12/12/2023). The excitement over ML is not unjustified. For instance, the infrastructure developed alongside ML (fast computing, robust coding frameworks, public accessibility of code and algorithms) is alone enough to celebrate (see, e.g., [15]). In an era of increasingly complex data, like data collected from wearable sensors or video monitoring in sports contexts, ML infrastructure has made managing and processing the data simpler, making what would otherwise be an extraordinarily complicated analysis task more accessible to scientists regardless of discipline.

But among the many high-profile successful deployments of ML (e.g. machine translation [16], strategy game players [17], airline route and pricing optimization [18], and special cases of automated assistance [19]) come many high-profile failures in deployment (see, for instance, examples in medicine [20–23]). Many of the failures occur because data in the real world are different from data used in the study [11]. One particular concern is that an algorithm might incorrectly leverage aspects of the data collection (e.g. image annotations from the investigator, settings for data collection instruments, etc. [24]) or even idiosyncrasies of the data itself (complex, happenstance associations that would otherwise be discounted by the investigator). These failures should alarm the scientific community, especially in fields like sports science and sports medicine that impact quality of life. On the one hand, we do not want to miss out on the promise of supervised ML to accelerate the pace of discovery in sports science and sports medicine (e.g. [25]). On the other hand, we do not want our work to negatively impact the individuals we are trying to help.

### Data Analysis in the Hypothetico-Deductive Framework

At this point, the reader may note that statistical models are also not immune from issues stemming from data quality or idiosyncrasies in the data set. This is certainly true. There are two points worth mentioning. First, in the hypothetico-deductive framework, scientists build statistical models using information beyond that provided by the data. In the ideal case, a model is fixed prior to collecting the data (pre-registration attempts to operationalize this ideal) and is based purely on prior studies (potentially including a pilot study) and scientific domain/statistical theory. This allows an entire study, from data collection to statistical analysis, to be vetted by the relevant stakeholders before the study begins. In other cases where model building is in part subject to exploratory data analysis (though still largely informed by prior knowledge), the choices made in modeling are subject to scrutiny from the scientific community (that this does not always occur is less an indictment of statistical methods and more so of scientific culture), and such scrutiny occurs in the context of domain knowledge and statistical best practices.

Supervised ML algorithms, in contrast, can be described as using inductive reasoning [26], taking the supplied data as evidence and returning an algorithm that exploits associations in the data. (We point out a distinction between induction in ML algorithms and induction in science: the scientist can still use evidence that exists outside of the data when drawing conclusions using inductive logic.) Importantly, in a supervised ML algorithm the function $f\left(x\right)$ is constructed *in response* to seeing the data. Because supervised ML algorithms are domain-unaware beyond their access to the data at hand, they do not leverage constraints from domain knowledge. In an appropriate prediction context, this may be an advantage of supervised ML, since the best algorithms for prediction need not be those that are most faithful to the science [3, 4, 9]. But in the hypothetico-deductive framework, the inability to concretely encode scientific knowledge could lead to biases that both make the conclusions difficult to interpret and contextualize, and that could have otherwise been avoided. (Though we point out that not all biases can be avoided, even when using a statistical framework. Recognizing bias and correcting incorrect prior beliefs is a natural part of the ebb and flow of scientific knowledge [6] and underscores the importance of science being a collective enterprise. The goal is to reduce the prevalence and impact of bias when possible.)

Second, statistical methods are built on rich and robust mathematical theory that describe the behavior of the model and inference under different data settings and modeling choices. Surprises in the statistical model (e.g. a negative sign on a coefficient that should be positive, or a known-to-be important variable that appears not to be significant) can be mathematically examined in the context of the entire model (e.g. potential presence of Simpson’s paradox or multicollinearity), in the context of domain knowledge (e.g. not including important interactions or controlling for important variables), and in the context of data collection (e.g. “the experimental design led to oversampling skilled runners, which is known to affect the significance level of the surprising variables”). Supervised ML algorithms tend to be more opaque, making algorithm examination more difficult. An inability to fully evaluate the algorithm means an inability to even recognize potential concerns prior to seeing the algorithm fail (e.g. an algorithm that uses a variable counterintuitively), an inability to contextualize an algorithm with respect to previous studies (e.g. an algorithm that, unbeknownst to the user, fails to include traditionally important interactions), and an inability to leverage the algorithm when thinking about issues in data collection.

### ML in Sport Science

Systematic reviews show interest in using ML in sports applications [27–30] but using ML in a sport science context can be challenging [28, 31, 32]. Similar findings hold in other studies of clinical prediction models (e.g. [33]). Further, when ML algorithms are used, sound methodology and high-fidelity interpretation of the results can prove challenging. A 2021 systematic review [34] evaluated 11 articles that deployed ML to predict injuries in sport. ML methods utilized in the reviewed articles were tree-based ensemble methods (n = 9), support vector machines (n = 4), and artificial neural networks (n = 2). They found that injury predictive performance ranged from poor (AUC = 0.52) to strong (AUC = 0.87). The review focused on the mechanisms used to build the 11 ML algorithms and noted a promising future for ML in sports medicine. The field of sports medicine is familiar with scales which evaluate a study’s methodological quality, its translation to evidence-based medicine and clinical utility. While there are many scales available for use, the authors of this review graded the included articles using the Grading of Recommendations, Assessment, Development and Evaluations (GRADE) scale [35]. They found the articles were of very low to moderate quality.

The methodology and findings of these 11 articles are focused on the deployment of ML and not necessarily on the advancement of patient care and clinical practice, yet they are published as such. Notably, the reviewed articles were unable to directly contextualize their resulting algorithms with respect to the theoretical foundations of injury, since either the algorithms were too complex (even if “interpretable,” like a decision tree) or variables or interactions deemed to be of potential clinical relevance were not able to be interpreted with respect to the entire model. Athletic injuries are complex, dynamic, and individual processes [36–38]. When domain knowledge is left out of the model-building and checking process, algorithms with high performance on paper are likely to fail when deployed in the real world (see, e.g. [11] and [39]). Further, algorithms may suffer in practice due to lack of clinical utility and potential barriers to implementation. For example, a model may reflect that minute differences in range of motion are key factors in injury prediction, yet such differences may not have clinical utility if these differences do not equate to an established minimally important difference threshold and would not be likely to influence clinical practice. As another example, an algorithm may detect that biomechanical patterns begin to shift during a very specific stage in an endurance trail run, yet practically there are key barriers to addressing movement deviations in the field where clinicians are often not present and would not be readily able to perform clinical interventions. The ability to deploy and hypertune an algorithm to a data set should not be mistaken at face value for high-quality medical research.

Before proceeding, it is worth noting that the field of ML is vast and can be divided into several overlapping subdomains. In this article, we focus on the role of supervised learning in the hypothetico-deductive framework for the reasons provided in the first paragraph of Sect. “Supervised Machine Learning”. We now provide a brief overview of a few of the ML subdomains most relevant to this paper. It is our belief that the most productive approach to understanding how ML can be used in sport science (and science more broadly) is to consider each ML subdomain/scientific framework pairing separately due to the broad scope of the field of ML.

### The Field of Machine Learning can be Divided into Several Overlapping Subdomains. We Briefly Describe Six Subdomains that are Most Relevant to this Paper

Supervised ML [7] is the most relevant subdomain to this paper. In supervised learning, the data consists of a set of predictors and an outcome variable or variables. For instance, *injury* can be the outcome of interest, and variables describing an individual (physical characteristics, workload, sport, strength, etc.) the predictors. In many time series scenarios, the observation at, say, time $t+1$ can be the outcome, and prior observations the predictors. Supervised ML is both powerful and widespread. For example, large language models (often classified as “generative AI”) use supervised ML for training (e.g. predict a word given its context).

Unsupervised ML [40] does not use an outcome for training. Rather, it seeks structure in the set of “predictors” alone. Among other tasks, unsupervised learning can be used for clustering, which in the hypothetico-deductive framework can be examined for potential insight [41, 42]. However, like supervised ML, potential insights should be treated with caution. That structure may exist in the set of predictors does not imply that the structure is *meaningful* structure. Importantly, in unsupervised ML, a chosen metric is optimized to find structure. There is always a structural configuration that maximizes the metric, but the “best” configuration from a numerical standpoint does not imply a “good” configuration from a scientific standpoint. But even if a strong structural configuration is found in the data, because it was not optimized with respect to a particular prediction task of interest (e.g. predicting injury), there is no guarantee that the found structure aligns well with the scientific task (for instance, college aged runners could be grouped in part according to which set of team-issued training shoes they prefer, but this grouping would in many cases be irrelevant and distracting). Relatedly, unsupervised learning can be sensitive to data preprocessing (variable selection, standardization, outlier removal, etc.) [43], which may increase the risk of unwitting data snooping and confirmation bias. Caution should be taken when interpreting and reporting results from an unsupervised ML algorithm. We leave a full exploration of these ideas to a separate manuscript.

Semi-supervised learning [44] is a large subdomain of machine learning that combines supervised and unsupervised learning. Semi-supervised learning uses both labeled and unlabeled data, typically with many more unlabeled than labeled observations. In large part, semi-supervised learning aims to either leverage the unlabeled data to improve prediction in a supervised algorithm, or leverage the labeled data to improve the performance of an unsupervised algorithm. We do not explore this further, but believe that the recommendations made in this paper can be applied when using semi-supervised learning to improve a supervised learning algorithm inside the hypothetico-deductive framework. In the other direction, recommendations from a treatment on integrating unsupervised learning in the hypothetico-deductive framework can be applied.

Causal ML [45, 46] is a subdomain that leverages ML in causal inference. Typically, causal ML involves applying ML to relevant parts of the statistical causal model. For instance, in matching, an ML algorithm might be used to estimate propensity scores or inverse probability of treatment weights. Causal ML is a relatively new field, and is most convincingly applied in conjunction with well-established statistical approaches. Further, in most specifications of causal ML, a particular treatment is required to be specified. Because we are interested in using ML for detecting potential “association” in this paper, we do not delve further into causal ML in this paper.

The last two subdomains of ML we discuss here are explainable ML and interpretable ML. The exact definitions of “explainable” and “interpretable” are somewhat slippery, and in this paper we follow conventions laid out in [10]. Rudin defines explainable ML to be post-hoc techniques applied to black box algorithms to attempt to understand how they are using the data to make predictions. She stresses, however, that explainable techniques are often misleading and incorrect. For the purposes of this article, explainable ML presents a reasonable technique for gleaning potential insights, but as with any other ML subdomain, potential insights should be scrutinized from a scientific lens to avoid wasting time and money. The authors of [47] advocate that in many situations, interpretable ML is preferable to explainable ML. Interpretable ML involves using models that are natively interpretable (e.g. decision trees, logistic regression, and LASSO). They argue that in most situations, a “black box” algorithm can be exchanged with an interpretable algorithm without loss of performance. It should be noted, however, that the performance of interpretable ML can depend on successful feature engineering (e.g. transformations and (nonlinear) combinations of variables) that are both interpretable and have predictive power. Such feature engineering can be challenging. Further, there is an increased risk of what we call in this paper “metric hacking” (see below) when features are constructed and added to improve the predictive power of the algorithm (rather than being scientifically justified). The pipelines proposed in this paper can be useful for both scientists and reviewers for protecting against the risks of metric hacking.

This manuscript aims to provide a useful framework for understanding how studies in sport science that exist in the hypothetico-deductive framework can leverage the power of supervised ML while protecting against failure. The key lies in understanding how and when supervised ML is or isn’t compatible with hypothetico-deductive reasoning. **We argue that using supervised ML in the hypothetico-deductive framework is most appropriate for exploratory work as long as any potential insights gleaned are isolated from the scientific process except through carefully controlled hypotheses that can be scientifically justified outside the algorithm's context**. Along the way, we provide high-level explanations and a useful metaphor for supervised ML. We aim to convince the reader that supervised ML can be an asset to the sports science and sports medicine community when used with care, and we provide examples of how to do this. At the end, we provide tips that enable editors, reviewers, and readers to evaluate and critique studies that rely on supervised ML.

Note that we use “statistics” and “supervised machine learning” (or often “models” and “algorithms”) in this paper to distinguish between two common approaches to working with data. Of course, these are not the only ways to distinguish between them and are not necessarily the most precise, but we use them here to match common parlance in data analysis.

## Leveraging Supervised ML in a Hypothetico-Deductive Framework

We first discuss ways in which supervised machine learning *can* be used in a hypothetico-deductive context and provide three case studies as examples. In Sect. “Discussion: Machine Learning in the Hypothetico-Deductive Framework”, we lay out our justification for why a principled approach to utilizing supervised ML is required.

### Supervised Machine Learning is of use to Sports Medicine Researchers, but Care Must be Taken as to How it Enters the Hypothetico-Deductive Framework

So how can we use supervised machine learning safely? To answer this question, we revisit a statistical stronghold, exploratory data analysis (EDA). John Tukey lays out our path forward in the introduction to *Exploratory Data Analysis* [48]. He says,

“As all detective stories remind us, many of the circumstances surrounding a crime are accidental or misleading. Equally, many of the indications to be discerned in bodies of data are accidental or misleading. To accept all appearances as conclusive would be destructively foolish, either in crime detection or in data analysis. To fail to collect all appearances because some—or even most—are only accidents would, however, be gross misfeasance deserving (and often receiving) appropriate punishment.”

Importantly, given the unknown role a piece of evidence may or may not play in our investigation, he goes on to say, “Exploratory data analysis can never be the whole story.”

When used for knowledge discovery, supervised machine learning is very much like the circumstances in the crime scene described by Tukey— findings could be spurious or not meaningful for our scientific investigation. But occasionally, it might provide a gem that breaks the case wide open. Supervised ML can supply some directions to pursue, **but the scientist working in the hypothetico-deductive framework must always decide if they are promising directions for future study via hypothesis-driven investigation.**

**Fig. 1:**
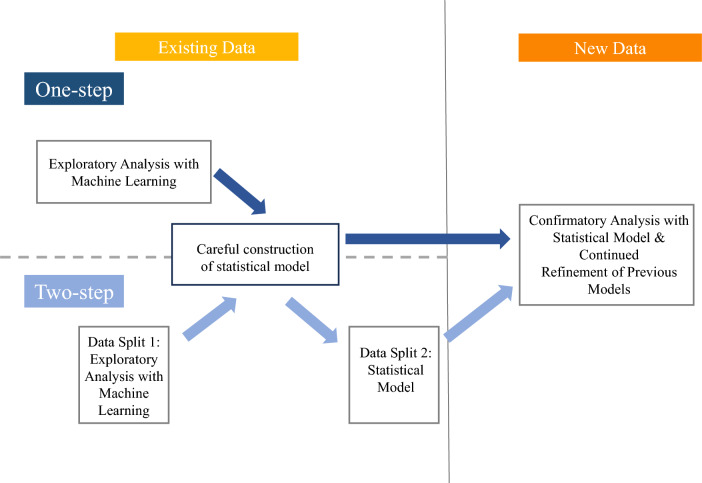
Supervised machine learning methods flow chart. The left side of the flow chart depicts the parts of the processes (“one-step” (top) and “two-step” (bottom)) pertaining to existing data. The processes then flow to the right side which tests a hypothesis on a novel data set

As with all EDA, there are several ways to proceed with the information we glean. An important consideration is separating EDA from confirmatory analysis (confirmatory testing for a trend on the same data we used to discover that trend artificially inflates the probability of a “discovery”). We outline two potential methods here (see Fig. 1). Both methods are ideally used as mechanisms to generate hypotheses for future studies. If results from these EDA methods are deemed interesting enough to disseminate in their own right to spur investigation by other labs, the status of the result (e.g. exploratory) should be clearly indicated, and the details of the EDA should be described in full to provide as much context for the degree of plausibility for the idea established by the exploratory analysis.

The two methods we propose, called “one-step method” and “two-step method” share a common goal of using supervised ML to generate suggestions for hypotheses that can be tested with future studies. These methods are exploratory because they apply supervised ML in a context for which it was not designed (i.e. for providing potential explanatory insights for further study, rather than for pure prediction). However, as with the detective in Tukey’s example, we might find some of these suggestions do seem reasonable and hold up against further data collection efforts. Other suggestions we may consider and then discard, incurring minimal to no expense.

The two-step method is an extension of the one-step method for cases where enough data is available and the scientist wants additional safeguards in place before designing a new experiment and collecting new data (e.g. when data collection is hard or expensive). With both methods, the steps are: 1) use supervised ML algorithms to provide suggestions on potential future directions; 2) after careful consideration of the suggestions in the context of the particular domain, construct a hypothesis to be tested if warranted; and 3) build statistical models or approaches that integrate the hypothesis into existing domain knowledge, and run a *“sanity check”* of the model on the current data set (note that this is not always possible if the relevant data were not collected (see Sect. “Features Predictive of Overuse in Elite Football (soccer) Athletes”)) to ensure that the hypothesis *would have been* confirmed on the current data set (see next paragraph for important cautions), given the proposed model. Such integration could be, for instance, constructing a plausible statistical regression (controlling for appropriate variables–see Sect. “Explainable and Interpretable Machine Learning Provide (Some) Access to Understanding how an Algorithm Works, but Still Pose Issues in the Hypothetico-Deductive Framework” for an explanation for why using an “interpretable” ML approach like LASSO out of the box does not necessarily yield a scientifically plausible regression function) that is consistent with prior studies (or challenges them in scientifically justifiable ways) and whose p-value for the coefficient[s] related to the relevant variable[s] is [are] significant.

As it can be difficult to understand how exactly a variable is used in an supervised ML algorithm (see Sect. “Discussion: Machine Learning in the Hypothetico-Deductive Framework”), and because good prediction models (recall, the historical focus of ML) are not necessarily scientifically faithful models (see Sect. “Introduction”), step 3 guards against wasting time and money on an experiment to test a hypothesis that may rely on scientifically irrelevant information or may be too difficult to test given the current state of knowledge of the field, since we may not yet be equipped to understand the relevant nonlinearities and interactions that are important to the expression of the variable of interest. The idea is, if we are unable to confirm the hypothesis using suitable statistical models or methods in the overly optimistic setting of using a data set on which the supervised ML algorithm actually identified the “suggested” hypothesis, we should be wary of hoping that we can recover signal in a way that can be contextualized in domain knowledge on a novel data set. We caution here that step 3 should be *strictly* a sanity check. In general, step 3 is called “double dipping” and is not typically a good practice. This is why having the safeguard of designing a novel experiment or study is crucial for protecting against false discoveries (see the next paragraph for reasons why a novel study is also important while using the two-step method). Restating to emphasize, the idea behind step 3 is that if the investigator cannot propose a suitable statistical model that also finds a particular variable or feature to be important *on the same data on which the supervised ML algorithm found it to be important*, then the investigator is unable to propose *how* the feature is important in the context of domain knowledge.

As mentioned, in some cases testing the hypothesis on the existing data set may not be possible. See Sect. “Features Predictive of Overuse in Elite Football (Soccer) Athletes” for an example where the totality of the information gleaned from the ML algorithm suggests integrating a common physiological measurement that was nonetheless not collected in the data set under study. In this case, proposing a plausible statistical model or approach is still useful as it provides a platform for the investigator and other scientists in the community to vet the plausibility and potential impact of a proposed future experiment. Whether or not the proposed model can be run on the existing data set, construction of a suitable statistical model or method provides a mechanism for the scientist and scientific community to examine the plausibility for the success of a hypothesis in a novel experiment.

The two methods differ in that the two-step method reserves a subset of the data that can be used as an intermediate test for the hypothesis. While both methods encourage identifying a scientifically justifiable statistical model or method that can be critiqued in the context of domain knowledge and that could serve as the basis of a test in a novel experiment, the two-step method allows the hypothesis to be partially tested on an “unseen” data set (at least unseen with respect to the hypothesis of interest) yielding additional strength to the plausibility of the hypothesis. While the “two-step” method has similar features to the training/testing data split common in machine learning, the motivation is different, and thus the two should not be confused. In the training/testing split, the goal is to avoid overfitting to the data in a prediction context—we recommend adhering to ML good-practices like using a training/testing data split in the *first* step of the “two-step” method as a protection against overfitting. (Supervised ML requires validation. Internal validation includes data splitting procedures like the training/testing data split, and it is often designed so that the two subsamples ideally come from the same distribution. External validation applies a prediction algorithm to a truly independent set of observations (e.g. from a different hospital). After deployment of a supervised ML algorithm, it remains critical to monitor the success of the algorithm [9]). The second step of the two-step method is intended as a check on the *statistical* model *inspired* by the supervised algorithm. In particular, it only partially serves to protect against overfitting of the supervised algorithm. More importantly, it serves as a check on the *translation* of the insights from the supervised algorithm to a potential statistical algorithm that is properly situated with respect to prior knowledge.

**We caution that when using the “two-step” method, the scientist must take care when interpreting the statistical analysis on the second part of the data, and not be lulled into the impression that it presents an independent analysis.** The chief concern is that these data were not collected with the specific hypothesis in mind, and thus issues that are often mitigated in carefully and specifically designed data collection cannot be eliminated. A full investigation requires the collection of new data specific to the hypothesis under consideration. Further, it can be tempting to iterate between running and interpreting ML algorithms and constructing statistical methods until a significant finding can be established. This should be avoided. In theory, once a statistical model or method has been constructed and evaluated, the data set should be considered unusable. Otherwise there is a risk of overfitting the data set, weakening the plausibility of the hypothesis. This is why the investigator must still do the hard work in considering how the results (i.e., suggestions) from the ML algorithm fit into the domain science.

The two-step method is also useful when a new data collection method becomes available in which there is a lower barrier for data collection, like wearable sensors, but where the data are not as immediately interpretable (e.g. because of lack of experience with the method) and the specific conditions under which the data are being collected are unknown. In this scenario, ML algorithms can be used to construct features of the data that provide predictive power. While interpreting the features and understanding exactly how they are used in the ML algorithm might be challenging, it may be possible to use the information gleaned from the algorithm to construct a feature that *is* interpretable and scientifically meaningful. The second step of the two-step method can be used to isolate this interpretable feature, place it in the context of known relevant variables, and test it for possible significance. Then, the investigator can design and pre-register a new study (if appropriate and expected in the investigator’s field, e.g. [49] [50]), and collect new data to formally test the hypothesis.

### Case Studies

Here we offer three case study examples based upon existing data to exemplify appropriate uses of supervised ML in sports science contexts. The first two cases show examples of the “two-step method” while the third case shows an example of the “one-step method.” In the first case, the two-step method led to a plausible insight, while in the second case translating insights from the ML algorithms used was challenging. In the third case, we highlight how one might use the one-step method to propose a potentially plausible study when the appropriate data is not available to provide additional “checks.” In this case, it would be a good idea to leverage colleagues in the sport science community to vet the promise of a new study. These examples provide a framework for future data collection and analysis planning to appropriately implement ML while mitigating risks (see Sect. “Discussion: Machine learning in the hypothetico-deductive framework”).

#### Feature Analysis of Runners with Exercise-Related Lower Leg Pain (ERLLP, or “Shin Splints”)

Historically, running gait assessments on injured runners have been confined to laboratory settings to examine specific biomechanical features that contribute to running-related injury etiology. Given that these assessments encompassed a finite number of steps in the gait cycle, more straightforward statistical analyses were generally accepted to model these data. However, we implemented a study design using wearable technology with injured (ERLLP patients) and uninjured runners to examine movement profiles in natural running scenarios over a week of training [51]. This approach allowed us to gather more ecologically valid field-based data, while simultaneously exponentially increasing the amount of running data (biomechanical measures from every step of every run over 1 week). Statistical analysis approaches that collapse multiple observations into average trends would overlook the nuanced gait patterns over time and their potential association with ERLLP. For example, simply taking an average of a biomechanical measure from thousands of steps across one run or multiple runs across a week would mask temporal stride-to-stride variations that may have meaningful clinical implications for injury progression. We additionally were interested in determining what biomechanical patterns may be evident during outdoor running that would not necessarily be detected during indoor running due to the controlled laboratory environment (i.e., holding constant speed on a treadmill). In order to expand the scope of potential nuances in gait patterns that we could initially consider, we implemented a supervised ML analysis on sensor-derived biomechanical data as an initial exploratory approach to suggest directions for subsequent analyses.

Using the “two-step method,” we ran a supervised ML analysis on half of our data set (32 of 64 participants) to elucidate defining biomechanical features among ERLLP and healthy runners. We input all sensor-derived biomechanical outcomes into the TSFresh Python program to compress our multivariate time series data to a set of features that were no longer time-dependent. The program takes a comprehensive approach by extracting over 1200 features per variable, including standard summary statistics (e.g., minimum, maximum, mean, standard deviation) and more advanced statistics (e.g., complexity, entropy, autocorrelation analyses) across varying lengths of data segments [52]. The program then returns a summary list including features of interest that were adept in differentiating the injured and uninjured running groups.

This supervised ML approach reflected that features related to contact time, the amount of time in milliseconds that the foot was in contact with the ground, was larger in magnitude and more variable for the ERLLP group compared to the healthy group. These findings gave us direction for analyzing the remaining data set via analysis of covariance and approximate entropy of contact time metrics, which was beneficial given the number of biomechanical metrics that can be gleaned from the wearable sensors. We also turned to approximate entropy and coefficient of variation analyses given that the program returned contact time variability as a feature of interest, which was unlikely to have been part of our initial data analysis approach were it not for the supervised ML results. By using the supervised ML analysis as an initial “guide” to assessment, we were better able to hone our subsequent analyses for assessing outcomes of interest in our clinical population.

#### Feature Importance Within Athlete Monitoring Data on a Collegiate Basketball Team

The field of athlete monitoring in team sports is not new, however, the evolution and subsequent commercialization of technology marketed to sports science and medicine practitioners has revolutionized the type, amount, and frequency of data that can be collected *in-situ*. From sensors that track on-court workload and force plates that test measures of strength and athletic readiness to wearable sleep monitoring, biomarkers, and athlete self-report measures (ASRM), the amount of information available to practitioners on a daily basis can seem innumerable compared to where the field was a decade ago.

Supervised ML has powerful predictive capabilities that have shown potential in sports analytics [53] and in athlete monitoring and injury prediction [54, 55] though leveraging supervised ML in the context of athlete monitoring and injury prediction is not always straightforward [27, 56]. There are many reasons for the poor prediction outcomes in injury including injury reporting, the individualized progression of injury, data collection methods, and the importance of temporal precedence in the study of overuse injury. To attempt to leverage the strength and flexibility of supervised ML for this data set, the “two-step” method was deployed.

The data were captured on 13 male collegiate basketball players over the off- and pre-season [57]. The full data set consisted of external load variables taken from wearable accelerometer devices and weight room tonnage, readiness variables captured from countermovement jumps conducted on force plates, and ASRMs collected from a survey instrument. The data were split along the off-season and preseason divide. A suite of supervised ML algorithms (random forest, ordinal forest, and support vector machine) were run on the off-season data, each classifying athlete self-reports of muscle soreness. Mean square error was used to determine best performance between the 3 algorithms. From the best performing algorithm on each variable grouping (external load, readiness, and ASRM), one variable was chosen, determined in part by an analysis of feature importance and in part through its plausibility of association with soreness, and was selected for use in the analysis of the preseason data. The chosen variables were: number of decelerations during on-court basketball activities, force at peak power during a countermovement jump, and self-reported physical performance capability (PPC). On the latter half of the split, data from the pre-season (not seen by the ML algorithms) were used to attempt to construct a logistic regression to predict above or below average soreness using the variables. Only the PPC binomial logistic regression was statistically significant. The model found the odds of having above average soreness decreased by 57% for every point increase in self-reported PPC (OR = 0.57, 95% CI 0.32 to 0.97, *p* < 0.05).

The next step in this case study would be for an investigator to think carefully about the clinical utility of PPC with respect to basketball and in the context of the team practice structure. If it is found to be potentially useful, the investigator could carefully design a new study and collect new data (e.g. the following season/pre-season) to further assess the association of PPC and soreness, and assess the practicality of making meaningful clinical decisions using PPC. While decelerations and force at peak power were not found to be significant, we note that the null result does not imply that they are indeed not significant or important in the context of muscle soreness. They may not be, but it is also possible that we simply do not understand at this point how they play a role in soreness, or at least not well enough to produce a useful statistical model. If we believe that they are indeed important (say, anecdotally) then it is worthwhile spending more time thinking carefully about how they may be important. But simply overlaying a complex model (e.g. supervised ML) to a complex problem is not a solution. As supervised ML continues to expand and become more accessible to non-technical users, practitioners and researchers should cautiously and thoughtfully interpret supervised ML results and verify findings and associations using well understood statistical analysis techniques.

#### Features Predictive of Overuse in Elite Football (Soccer) Athletes

Our third case study shows the “one-step” method and comes from the literature. The authors in [58] follow a cohort of elite youth football (soccer) athletes throughout one season of play. Using features measured at the beginning of the season (both anthropometric measurements as well as tests of coordination and fitness), the authors predicted if athletes would get injured during the season, and if an injury was acute or overuse. Using the extreme gradient boosting algorithm, the authors showed promising predictive results.

We focus on the author’s interest in distinguishing between an acute and an overuse injury. After examining feature importance measures, the authors noted that, “After [predicted] age at [peak height velocity], the most important variable is performance on the moving sideways task, followed by the 20 m sprint time and the t-test performed with left turns.” (The t-test performed with left turns should not be mistaken for the statistical t-test.) As the authors note in their practical applications section, no one feature is highly predictive of injury, and they encourage users of their multicomponent approach to focus on broader profiles to assess injury risk.

However, we can take the top features from the ML algorithm– moving sideways task, 20 m sprint, and agility t-test with left turns– and ask, are there any underlying physiological or contextualized factors that were unaccounted for that could be associated with better scores in those tests, and that also seem like reasonable differentiators of overuse/acute injury? For example, the top three features identified each represent constructs of agility or speed. An underlying physiological factor that was unaccounted for in the study and that may be associated with better scores in these 3 measures is “hip power.” The results from the ML analysis, coupled with domain knowledge specific to functional performance, could be used to justify the rationale for developing a new hypothesis-driven study that would aim to assess the role of performance on a test of hip power in predicting football (soccer) players who may be at greater risk of acute versus chronic injuries. Further, note that “standing broad jump,” which is closely related to hip power, is a low ranking variable according to feature importance. This could be because information about hip power in the standing broad jump is redundant in the presence of the top three variables. Dropping those variables, rerunning the algorithm, and assessing the rise in rank of standing broad jump importance could provide further justification for the hypothesis-driven study when an increase in rank is substantial.

In the next section welay out argumentation showing the need for principled frame works for carefully incorporating machine learning into a scientific pipeline.

## Discussion: Machine Learning in the Hypothetico-Deductive Framework

### Historically, Machine Learning has been Used for Prediction and Classification. Using Machine Learning in the Hypothetico-Deductive Framework Involves Repurposing and should be Approached with Caution

As previously mentioned, both statistical models and supervised ML algorithms aim to find a function to satisfy the equation $y=f\left(x\right)+\epsilon$. What is the nature of the function $f\left(x\right)$? It depends on the context. For instance, we may suppose that there is a well-specified “data generating process” (e.g., a function produced by “nature”), and our goal is to recover that function. Recovery here means that we ultimately want to know which variables, nonlinearities, and interactions are included in the true data generating process. Because nature is complex, we may never achieve perfect recovery, but can hope to achieve a *useful* recovery. In a sense, the search for $f\left(x\right)$ is an attempt to answer “how” variation in $x$ is linked to variation in $y$. Alternatively, we may not care about the true data generating function (or may not believe one exists). Rather, we simply want a function that makes good predictions of $y$ using $x$ (and instead of talking about the “true” $f\left(x\right)$ we can talk about the “optimal” $f\left(x\right)$). Because in this case we need not necessarily reason about the *way* in which variation in $x$ space is mapped to variation in $y$ space, we may be happy to work with functions that are difficult to unpack and describe. In fact, we do not necessarily require a “human readable” function at all: a nonparametric approximation to $y={x}^{2}$ suffices, since understanding that “$y$ varies according to ${x}^{2}$” is not necessary. Here, the search for $f\left(x\right)$ is an attempt to answer if variation in $x$ “can” be used to prediction variation in $y$. Historically, statistics has concerned itself a great deal with “how.” Historically, ML has concerned itself almost exclusively with “can.” See [8, 59] for an interesting discussion of the “two cultures” (model and algorithmic) of data analysis.

A major difference between statistical and ML approaches is how they constrain functions. In classical statistics, the constraint is on the functional form (e.g., which variables are included or the presence of nonlinearities and interactions) and is the purview of the scientist. The functional form is chosen so as to sufficiently isolate the effect under study while controlling for additional sources of variation that could confound the results. In ML, on the other hand, the constraint is on how *flexible* the function is allowed to be through choices of algorithm and algorithmic settings (even if the scientist chooses *which* variables to use, the scientist has less control over *how* they are used). The ML algorithm starts with the ability to approximate many different functional forms and finds the “best” function through numerical optimization routines that balance predictive accuracy with constraints on flexibility.

This difference in constraints has implications for our ability to contextualize results with respect to prior domain knowledge. In a hypothetical scenario involving 30 variables from an experiment using a wearable sensor, a scientist initially constructs a linear model with 7 variables (2 under direct study, 5 for known variation control). Later, for fun, the scientist employs an ML approach using LASSO—often considered an ML technique despite being developed by statisticians [14]—on all 30 variables, resulting in a model with 10 variables, 4 of which overlap with the initial statistical model. The challenge in interpreting the ML model arises from the fact that variable selection is based on numerical optimality rather than explicit scientist choices. This complexity is exacerbated when ML algorithms allow nonlinear combinations (e.g., random forest). In order to interpret the LASSO with respect to the statistical model, and the hypothetico-deductive framework more generally, the scientist must (a) determine what variation in the ML algorithm serves as a proxy for variation in the statistical model, keeping in mind that association with a variable in the statistical model might be nonlinear and involve multiple variables, (b) determine how to make sense of variation in the ML algorithm that is not included in the statistical model (ideally in an interpretable way that can be concisely communicated to the community), and (c) determine how to make sense of variation captured by the statistical model that was left out of the ML algorithm.

Like classical regression, the prediction surface of the LASSO is a high-dimensional plane called a hyperplane. We will simply call this a “plane.” The plane has a slope of zero along the directions of the variables (each variable corresponding to an axis) that have been “dropped” by the LASSO. As mentioned before, the difference between the LASSO and a classical statistical regression is that, in a classical statistical regression, the variables are pre-determined by the investigator. The estimation procedure in a statistical regression can wiggle the plane only along the specified directions. For LASSO, in contrast, all variables are in play, and the plane can be wiggled in all directions until a suitable (sparse) fit is found. The wiggles are motivated by numerical optimization. Some variables in the equation may be added at the exclusion of other more clinically relevant variables because of the complex multicollinearity that could exist in the data set; others may be added because they provide a suitable “adjustment” for a variable that *should have been* squared (for instance).

This toy example emphasizes two key points. First, statistical models undergo analysis in both $x$ space (considering the model's alignment with existing domain knowledge) and $y$ space (evaluating whether the variation in $x$ sufficiently explains the variation in $y$). In contrast, ML algorithms have traditionally been assessed only in $y$ space, focusing on their predictive capabilities rather than their adherence to domain dynamics [9, 59, 60]. Understanding how $x$ is utilized in predicting $y$ was *historically* not a primary concern for ML.

Second, when employing numerical optimization to constrain a function, an algorithm may be theoretically interpretable (as seen with LASSO, where coefficients can be interpreted as in linear regression) but not practically interpretable. This challenge is exacerbated when relying on interpretation mechanisms like “variable importance,” which reveals whether a variable is useful to the algorithm but obscures how it is used. It's crucial to note that being “useful to the algorithm” doesn't necessarily imply being “useful for *understanding*.” For instance, if two variables in a data set are unwittingly identical, the algorithm may use both, with the split of signal between them dependent on the random initialization of the algorithm. In the presence of redundant signal, there is no guarantee that an algorithm will leverage the most relevant source or even only one source.

For a useful theoretical discussion of the mathematical differences between models used for pure prediction and models used for explanation, see [61] and references therein. Another excellent discussion, though more technical, is in [62].

### Explainable and Interpretable Machine Learning Provide (some) Access to Understanding how an Algorithm Works, but Still Pose Issues in the Hypothetico-Deductive Framework

To extract meaningful information from an ML algorithm, we typically aim for high-level insights (e.g., “variable $x$ is heavily used by the algorithm”). However, caution is warranted when attempting to leverage these insights in the hypothetico-deductive framework, as such high-level information overlooks critical aspects of the algorithm's operation, such as *how exactly* a variable is used. It may not account for a variable being moderated in certain parts of the domain or the need for additional variables to accurately depict the relationship between the discovered variable and the outcome. Relying on such findings in the presence of these unknowns is akin to advising a clinician to use a specific metric for determining return-to-play status after injury without considering factors like an athlete's age and weight. This type of insight into an algorithm is subject to what we call in this paper *structural ignorance.*

Recently there have been efforts in the machine learning community to make this process of extracting information from algorithms easier, often called *algorithm explainability* (e.g. feature importance [63], partial dependence plots [64], Shapley values [65], etc.). The goals are three-fold. First, when purely making predictions (as a reminder, not the focus of this paper), knowing what features an algorithm is leveraging might make us *feel* better about the predictions being made. Second, having insight into what variables are important to an algorithm can help with algorithm diagnostics when predictions are wrong. Third, there is a hope that when algorithms can be explained, we might be able to translate that information into knowledge that can propel (clinical) decision-making.

Algorithmic explainability typically obscures how exactly a variable is used in an algorithm, and what (and how) other information is simultaneously being used (e.g. which variables are controlled for, and how) [10]. Thus, information extraction from an ML algorithm is problematic if it is directly treated as evidence. Instead, in the hypothetico-deductive framework, we should treat ML explanations as an offhand remark from a naïve student in an intro class who happens to say something that sparks our curiosity. We have no reason to think that the student based the remark on well-constructed scientific argumentation, but nonetheless, it resonated with us enough to warrant further scientific and statistical investigation (see [66] for an example where this happened after an otherwise naive question from a 10 year old student, and the backstory in *Popular Mechanics* [67]). Our case studies in Sect. “Case Studies” illustrate two methods for incorporating these “naïve suggestions” from the ML algorithm in our scientific workflow.

But what if we eventually get good at explainability? What if we get to the point where we *can* recover a fully human-readable (i.e. interpretable) function that relates the covariates to the outcome? Can we then run a highly flexible ML algorithm on our data in hopes that it will discover a previously unknown pattern that we can claim as new knowledge in our field? This is to some degree the hope of the *interpretable* ML approach. But we argue in the next section that, when working in the hypothetico-deductive framework, this is similar to p-value hacking when not treated strictly as exploratory.

### Achieving Full Explainability (or Interpretability) in ML Does not Mean that we Can Replace Statistical Methods with Supervised ML in the Hypothetico-Deductive Framework. Without Proper Checks in Place, this is Tantamount to p-value Hacking

Recall that supervised ML algorithms a) are inductive in nature, taking as evidence the supplied data set without additional scientific context, b) are constrained by numerical optimization alone, and c) are traditionally prediction algorithms, which typically perform better (and thus are optimal) when *not* constrained to be scientifically relevant. Importantly, the hypothetico-deductive scientific process isn’t reflected in the way the ML algorithm estimates a function. Rather, it leverages clever engineering to find an optimal (with respect to a chosen metric, numerical constraints, and best practices like cross validation) function approximation. Depending on the characteristics of the algorithm being used, in some cases this amounts to a search over many possible functions, while in others the path to the optimal function is more direct. But in either case the function fit is not optimized to be compatible or comparable to existing knowledge. This is not always a concern (e.g. in some pure prediction scenarios), since compatibility with the existing science may not be important (and certainly need not yield an optimal prediction algorithm). To see why this is problematic in the hypothetico-deductive framework however, consider how this would look if we found this same optimal function by hand.

Suppose Lab A runs an ML algorithm on their data (we can assume that all good practices were followed to avoid overfitting), and it returns a complex function, $f$, that encodes interesting patterns nobody in the field has yet considered. Lab B, on the other hand, hires an army of undergraduate and graduate students and trains them to try every imaginable combination of variables, functions of variables, and interactions of these functions of variables that they possibly can until they find the arbitrarily complex function that fits the data well (we are adapting the infinite monkey theorem [68] here). We can assume here as well that the students took care to avoid overfitting, like dividing the data into subsets in order to provide some notion of “replicability” of the model fit across independent data sets (e.g. find a model that does well on subset A, and then test that model to see if it still does well on subset B). Given enough time, the students in Lab B will eventually stumble on the function that was approximated by Lab A’s ML algorithm.

The difference between the two approaches is that Lab A used an ML algorithm to find that function, and Lab B took an approach that looks quite a bit like p-value hacking in the hypothetico-deductive framework, except instead of looking at p-values to decide when to stop, they looked at RMSE or some other metric indicating the success of a model fit to data. Let’s call this, more broadly, *metric hacking* (and note that p-value hacking is a specific version of metric hacking). Metric hacking is well known to be bad science in the hypothetico-deductive framework and played a major role in the reproducibility crisis [6]. So if Lab B is guilty of metric hacking, then what of Lab A?

We might hope that Lab A is doing something more than metric hacking (in the hypothetico-deductive framework) because they are using an algorithm, but as mentioned in Sect. “Introduction”, ML operates in an inductive framework taking the supplied data as its source of evidence, and simply optimizes the metric under consideration given the algorithmic constraints and best practices. In its most basic implementation, an ML algorithm could simply try every possible setting of its parameters (much like Lab B, though of course Lab B would be working strictly with human-readable functions). But as with the students of Lab B, this is incredibly inefficient. One catalyst of the rapid development of ML algorithms over the past few decades was figuring out how to efficiently find an optimal function from the same space of functions that the students in Lab B were asked to explore by brute force. The machine learning community has excelled in finding innovative, efficient ways to search this space even for highly complex algorithms. We reiterate that metric hacking as we have described it is an issue in the hypothetico-deductive framework, but not necessarily in other scientific frameworks, for instance where pure prediction is the primary goal.

### A note on Sample Size

One concern often mentioned with respect to ML is the need for a large sample size, though recent examples show promising results for building predictive models with small sample sizes under suitable protections against overfitting [69]. This is certainly a concern in much of sport science. Notably, small sample sizes may ultimately make ML unsuitable for some pure prediction problems, even when the scenario is otherwise favorable for applying ML. The *success* of leveraging supervised ML algorithms in the use cases we have proposed in this manuscript is also susceptible to sample size. However, potential failure due to problems with sample size is less of a concern in our case. This is because ML is used in our framework as only an intermediate tool. Any insights from the algorithm are subjected to scrutiny by the scientist for potential relevance, utility, and plausibility given the scientist’s knowledge of prior studies and theory in the domain (i.e. integration into the hypothetico-deductive framework). The sample size seen by the algorithm could be used as additional information to help the scientist decide how hard to try to test a potential insight that is on the edge of plausibility (e.g. “low sample size, maybe not worth it”). But ultimately, nothing prohibits a scientist from *not* pursuing an insight, despite a seemingly large sample size, if the scientist deems the insight to be implausible, not relevant, or not useful. In other words, an *uninteresting* suggestion need not be pursued, even if the sample size used by the proposing algorithm was large; an *interesting* suggestion can be pursued, whether it came from an algorithm using a smaller-than-ideal sample size, or a 10 year old.

## Conclusion

Supervised ML is a powerful tool for pure prediction problems that can have a useful role in the hypothetico-deductive framework when used appropriately. As with any method, care must be taken when deciding if ML is an appropriate tool for a particular problem. Much work by the community remains in determining for which problems ML provides a useful solution. In this manuscript, we examined the use of ML in the hypothetico-deductive framework, which is a common framework in scientific endeavors. We show that, while not directly applicable out of the box, supervised ML can have a role to play.

**Fig. 2:**
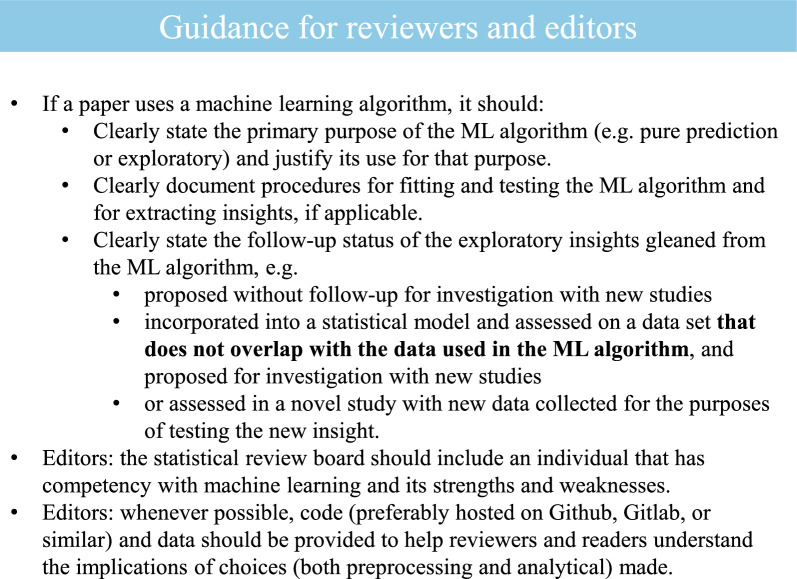
Machine learning guidance for reviewers and editors. Key points that reviewers and editors should be mindful of when assessing sports science manuscripts that introduce machine learning methods

One of the most impressive accomplishments of the ML community has been the democratization of these algorithms, making them easy tools to add to the scientist’s toolbox. Despite the fact that ML algorithms were designed for pure prediction, recent developments in explainable and interpretable ML have made their integration into the hypothetico-deductive framework tempting. Unchecked, this is reckless, especially in the clinical sciences. The two presented workflows, “one-step” and “two-step” (Fig. 1), offer direction to investigators in future research endeavors to leverage the flexibility of machine learning while still protecting against failure brought on by metric hacking. The workflows and discussion can also help editors and reviewers constructively critique manuscripts that use machine learning as primary analysis (Fig. 2). Most importantly, for sports medicine and sport science researchers, there should be an understanding that machine learning should not be approached and implemented in a silo. Collaboration between domain experts, statisticians, data scientists and engineers is encouraged. A team science approach to working with data fosters more robust results and advances science in a responsible manner.

## Data Availability

Not Applicable.

## Abbreviations

AI: Artificial intelligence
ASRM: Athlete self-reported measure
CI: Confidence interval
EDA: Exploratory data analysis
ERLLP: Exercise-related lower leg pain
GRADE: Grading of Recommendations, Assessment, Development and Evaluations
ML: Machine learning
OR: Odds ratio
PPC: Physical performance capability
